# Optimal time interval between hysteroscopic polypectomy and frozen-thawed blastocyst transfer: A retrospective study

**DOI:** 10.1371/journal.pone.0240882

**Published:** 2020-10-20

**Authors:** Yi-An Tu, Po-Kai Yang, Shee-Uan Chen, Jehn-Hsiahn Yang

**Affiliations:** Department of Obstetrics and Gynecology, National Taiwan University Hospital and National Taiwan University College of Medicine, Taipei, Taiwan; Universita degli Studi dell'Insubria, ITALY

## Abstract

The optimal timing of frozen-thawed blastocyst transfer following hysteroscopic polypectomy is an important and unanswered clinical question. In this study, we conducted a retrospective survey of cases from an infertility center at an academic hospital. We reviewed the charts of all patients who received in-vitro fertilization and frozen-thawed blastocyst transfers (FBT) at the center from January 2009 to November 2019. One hundred and two patients with prior diagnosis of endometrial polyp that were treated with hysteroscopic polypectomy before received their first FBT at the center were identified as cases. Patients without prior diagnosis of endometrial polyp, and who received their first FBT at the center were defined as controls. Controls were enrolled at a 1-to-1 ratio to the cases. The cases and controls did not show differences in baseline characteristics, endometrial thickness, or the number of good blastocysts transferred. The clinical pregnancy rates and live birth rates were similar. Regarding the optimal interval between polypectomy and FBT, a cut-off of 120 days was identified from the ROC curve. A stratified analysis showed that when FBT was performed within an interval of 120 days after polypectomy, there were higher biochemical pregnancy rates (73.2%, 45.2%; OR 3.3; *P* = .007) and clinical pregnancy rates (64.8%, 41.9%; OR 2.54; *P* = .032), when compared with intervals greater than 120 days. There were no significant differences in implantation and live birth rates. In conclusion, pregnancy rates following FBT in patients who had received prior endometrial polypectomy were comparable to pregnancy rates after FBT in patients without endometrial polyp. Subgroup analysis showed that an interval greater than 120 days between hysteroscopic polypectomy and FBT was associated with decreased pregnancy rates. Patients who wish to receive embryo transfer after polypectomy should wait no longer than 120 days.

## Introduction

Endometrial polyp is a frequently encountered abnormality of the uterine cavity, which may interfere with normal embryo implantation [1]. Resection of the endometrial polyp via hysteroscope has been shown to be an accessible, and beneficial intervention when performed prior to starting assisted reproductive treatments, such as intrauterine inseminations and in-vitro fertilizations (IVF) [2, 3]. Furthermore, when endometrial polyps are incidentally diagnosed during IVF treatments, we have shown that a freeze-all strategy followed by hysteroscopic endometrial polypectomy (HSC-P), and a vitrified-warmed embryo transfer is a viable option [4]. However, one question which has not been answered conclusively, is whether there exists an optimal interval between HSC-P and a vitrified-warmed embryo transfer.

Indirect answers to this question can be deduced from studies on endometrial healing. Studies on endometrial healing time following different hysteroscopic surgeries [5] have shown that the endometrium fully healed within 1 month of HSC-P. Other studies have pointed to a progressive increase in polyp recurrence following HSC-P, which becomes particularly pronounced after 1 year [6, 7]. These studies have provided a rough upper and lower bound for the suggested interval between HSC-P and the subsequent embryo transfer. However, these studies have not been able to provide data on the optimal timing for embryo transfers after HSC-P.

Studies on fresh embryo transfers following HSC-P have provided more direct evidence. Previously, cohort studies have compared embryos transfers after different intervals: less than 6 months to greater than 6 months [8]; 1 menstrual cycle, 2 to 3 cycles, to greater than 3 cycles [9]. These have generally shown no difference in pregnancy outcomes. However, it should be noted that these studies were performed on fresh embryo transfers, which may not be directly applicable to frozen-thawed embryo transfers (FET), given the purported difference in endometrial conditions between fresh and frozen transfers [10]. In addition, with the increased use of FET and freeze-all strategies [11, 12], this question has become pertinent to everyday practices. It is within this context that we aimed to assess the pregnancy outcome in patients who received FET following HSC-P. Furthermore, we would like to identify a possible optimal cutoff for the interval between HSC-P and FET.

## Materials and methods

### Subjects and ethics approval

In this retrospective study, medical records of women who underwent IVF or intracytoplasmic sperm injection (ICSI) cycles at the National Taiwan University Hospital between January 2009 and November 2019 were reviewed. This study was approved by the Institutional Review Board of the National Taiwan University Hospital on 10 June 2019 (NTUH-REC No. 201904081RIND). The consent form was not obtained because the data were analyzed anonymously and the characteristics of observational study.

All IVF/ICSI cycles from patients who received a first cycle frozen-thawed blastocyst transfer (FBT) were identified. Cycles with the following conditions were excluded: embryos from frozen-thawed oocytes, embryos cryopreserved for reasons related to malignancies, embryos from oocyte donation programs, and embryos that have received preimplantation genetic testing.

From the identified cycles, a study group (group 1) composed of women with any past diagnosis of endometrial polyps that had received treatment with HSC-P prior to FBT were enrolled. Women with incomplete records, such as absence of an office hysteroscope record prior to HSC-P or incomplete or absent surgical records due to surgeries performed at other hospitals, were excluded. Intervals (in days) between the HSC-P and the FBT were recorded. The controls were defined as patients from the same identified FBT cycles who did not have any prior diagnosis of endometrial polyp. Patient with any surgical intervention to the endometrium prior to FBT, which may have result in endometrial trauma, were also excluded. A 1-to-1 enrollment ratio of controls to cases was chosen, and a sample of the controls (group 2) were enrolled in an age-matched, random fashion. Briefly, the control group was sampled from the identified cases by first stratifying by 1-year age intervals, and picking every third case in each interval until the number of controls have been satisfied.

The age, gravida, parity, body mass index, number of endometrial polyps, infertility etiology, estradiol (E2) level on the day of ovulation trigger, the number of retrieved oocytes at ovum pick-up (OPU) cycle, endometrial preparation for the FET cycle, peak endometrial thickness, number of transferred embryos and number of good blastocysts, and pregnancy outcomes were recorded.

### Ovarian stimulation, oocyte retrieval, and embryo cryopreservation

Controlled ovarian stimulation was carried out using either a GnRH agonist short protocol, a GnRH agonist long protocol, a GnRH antagonist protocol [13], a progestin-primed ovarian stimulation protocol [14], or a switch protocol [15], as described previously. We monitored the cycles using serial folliculometry, serum E2, luteinzing hormone and progesterone. When the leading follicle(s) reached a diameter of 18 mm or more, and adequate estradiol levels had been achieved, 250–500 μg of HCG (Ovidrel®, Merck Serono, Darmstadt, Germany) was administered subcutaneously. For patients at risk of ovarian hyperstimulation syndrome, a 0.2 mg dose of Triptorelin (Decapeptyl®, Ferring, Kiel, Germany) was used instead. Transvaginal oocyte retrieval was performed under anesthesia 34–36 h later. The choice of IVF or ICSI depended on the quantity and motility of the male partner's sperms. Embryo culture and cryopreservation were carried out according to our standard lab protocols. Embryos were cryopreserved by slow-freezing prior to 2014, and by vitrification using the Cryotop method (Kitazato Supply Co., Fujinomiya, Japan) after 2014.

### Office hysteroscopy and hysteroscopic polypectomy

Office hysteroscopy was done in the follicular phase for diagnosis and localization of intracavitary lesions. The numbers of endometrial polyps were recorded. The procedure was carried out using a Hysterovideoscope HYF type V (Olympus Optical Co.), as described previously [4]. HSC-P was performed in the follicular phase of the menstrual cycle following the OPU cycle. The operations were exclusively performed by the same physician (J.-H.Y.), using a 12-degree resectoscope with an outer diameter of 8 mm (Olympus Optical Co.). All visible endometrial polyps were removed under direct hysteroscopic visualization using blunt force, via the application of the cutting-loop without diathermy.

### Frozen-thawed embryo transfer and follow up

For embryo transfer, we either used a natural cycle or an artificial cycle with hormonal replacements for endometrium priming. Blastocysts were thawed and transferred on the fifth day of spontaneous ovulation in natural cycles or the fifth day of starting progesterone in adequately estrogen-primed artificial cycles. Blastocysts were morphologically graded as good quality or poor using the Gardner grading system [16, 17]. Luteal phase support in natural cycles was performed using any combination of subcutaneous/intramuscular hCG (Pregnyl, Merck, Kloosterstraat, Netherlands), oral progesterone (Utrogestan, Besins, Ayutthaya, Thailand), and vaginal progesterone (Crinone, Merck, Industriestrasse Briseck, Switzerland). Luteal phase support in artificial cycles included the use of estradiol (Estrade, Synmosa, Hsinchu, Taiwan) with the same progesterone options as the natural cycles. We discontinued luteal phase support when a fetal heart beat was detected in the natural cycle, and after 10-week gestation in the artificial cycle.

Biochemical pregnancy was defined as a serum β-HCG level ≥20 IU/L on the 12^th^ day after embryo transfer [18]. Clinical pregnancy was defined as the presence of a gestational sac on ultrasound performed at 4 weeks after embryo transfer [19]. A birth after 24-week gestation that showed any sign of life, including breathing, heart-beat, umbilical cord pulsation or voluntary movements of the muscles, was considered a live birth [20]. Live birth rate and multiple pregnancy rate were calculated as the number of occurrences divided by the number of cycles initiated [21].

### Statistical analysis

Data are expressed as median with interquartile range (Q1, Q3). Because the data were not normally distributed, differences between the groups were compared using Mann-Whitney U test for continuous variables, and chi-squared test or Fisher’s exact test for categorical variables. A *P* value < .05 was considered statistically significant. On the basis of an alpha error of 0.05, a power of 80%, and a previous study comparing clinical pregnancy rate between women with FET after hysteroscopic polypectomy and fresh ET (63% versus 41%) without polyp [4], the estimated sample size was determined to be 80 per group at the minimum, for an enrollment ratio of 1-to-1. A receiver operating characteristic curve, or ROC curve, is created by plotting the true positive rate (TPR) against the false positive rate (FPR) at various threshold settings. Youden index is the sum of sensitivity and specificity minus one. The maximum value of the index is used as a criterion for selecting the optimum cut-off point. The area under the curve (AUC) is also calculated. All statistical analyses were performed with the PC version of the Statistical Analysis System (SAS version 9.4; SAS Institute) and the Statistical Program for Social Sciences (SPSS version 15; SPSS).

## Results

Table 1 shows the patient characteristics in the HSC-P group (group 1, n = 102) and the control group (group 2, n = 102). There were no differences in age, gravidity, parity, body mass index, or peak estradiol level during the OPU cycle. Patients in group 1 adopted freeze all embryos strategy due to following conditions: 65.7% having concurrent diagnosis of endometrial polyp during ovarian stimulation, 30.4% having high risk of OHSS, 2.0% having premature progesterone elevation, 1% with inadequate endometrial thickness, and 1% under patient request. The patients in group 2 did not have diagnosis of endometrial polyp and all underwent freeze-all embryos and then FBT for the following indications: 88.2% due to risk of OHSS, 3.9% due to premature progesterone elevation, 2.0% due to inadequate endometrial thickness, and 5.9% due to patient request. Apart from the diagnosis of endometrial polyp, the indications for embryo cryopreservation were similar between groups.

**Table 1:** Demographics of all patients.

	Group 1: Polypectomy	Group 2: Controls	*P* value^a^
Case no.	102	102	
Age (years)	36 (34, 39)	36 (34, 39)	1
Gravidity	0 (0, 1)	0 (0, 1)	.262
Parity	0 (0, 0)	0 (0, 0)	.828
BMI (kg/m^2^)	22.2 (20.7, 23.8)	21.2 (20.3, 23.9)	.096
**Endometrial preparation**			.652
Natural cycle	31 (30.4%)	34 (33.3%)	
Artificial cycle	71 (69.6%)	68 (66.7%)	
**Endometrial thickness** (mm)	10.7 (9.3, 12)	10.3 (9.5, 12)	.629
**ET no.**	2 (2, 2)	2 (2, 2)	.205
**Slow-freezing/vitrification**	18 (25.4%) /53 (74.6%)	9 (29.0%) /22 (71.0%)	.698
**Good embryo no.**	1 (0, 2)	1 (0, 2)	.532
**OPU cycle**			
Peak E2 (pg/mL)	2893 (1980, 4551)	3458 (2115, 5572)	.142
Oocyte no. retrieved	16 (12, 20)	19 (13, 23)	.046
**Indication for IVF/ICSI**			.778
Male	43 (42.1%)	37 (36.3%)	
Ovarian	21 (20.6%)	21 (20.6%)	
Tubal	11 (10.8%)	12 (11.8%)	
Endometriosis	6 (5.9%)	4 (3.9%)	
PCOS	6 (5.9%)	11 (10.8%)	
Unexplained	15 (14.7%)	17 (16.7%)	

Data are presented as median (Q1, Q3) and n (%). BMI: body mass index. E2: estradiol. ET: embryo transfer. No.: number. OPU: ovum pick-up. IVF: in vitro fertilization. ICSI: intracytoplasmic sperm injection. PCOS: polycystic ovary syndrome.

^a^*P* values were calculated using the Mann-Whitney U test for continuous variables, and Fisher’s exact test for categorical variables.

The number of oocytes retrieved in OPU is significantly higher in group 2 (19 vs. 16; *P* = .046). As for the proportion of natural versus artificial cycles, peak endometrial thickness, number of embryos transferred, method of cryopreservation and number of good quality embryos transferred, both groups demonstrated similar results. The pregnancy outcome of group 1 and 2 shows no significant difference in implantation rate (35.2%, 95%CI = 28.9%-41.5%, 33.7%, 95%CI = 27.3%-40.1%; *P* = .743), biochemical pregnancy rate (64.7%, 95%CI = 55.4%-74%, 60.8%, 95%CI = 51.3%-70.3%; *P* = .664), clinical pregnancy rate (57.8%, 95%CI = 48.2%-67.4%, 59.8%, 95%CI = 50.3%-69.3%; *P* = .887), live birth rate (47.1%, 95%CI = 37.4%-56.8%, 43.1%, 95%CI = 33.5%-52.7%; *P* = .673) or multiple pregnancy rate (7.8%, 95%CI = 2.6%-13%, 5.9%, 95%CI = 1.33%-10.5%; *P* = .783).

**Fig 1:**
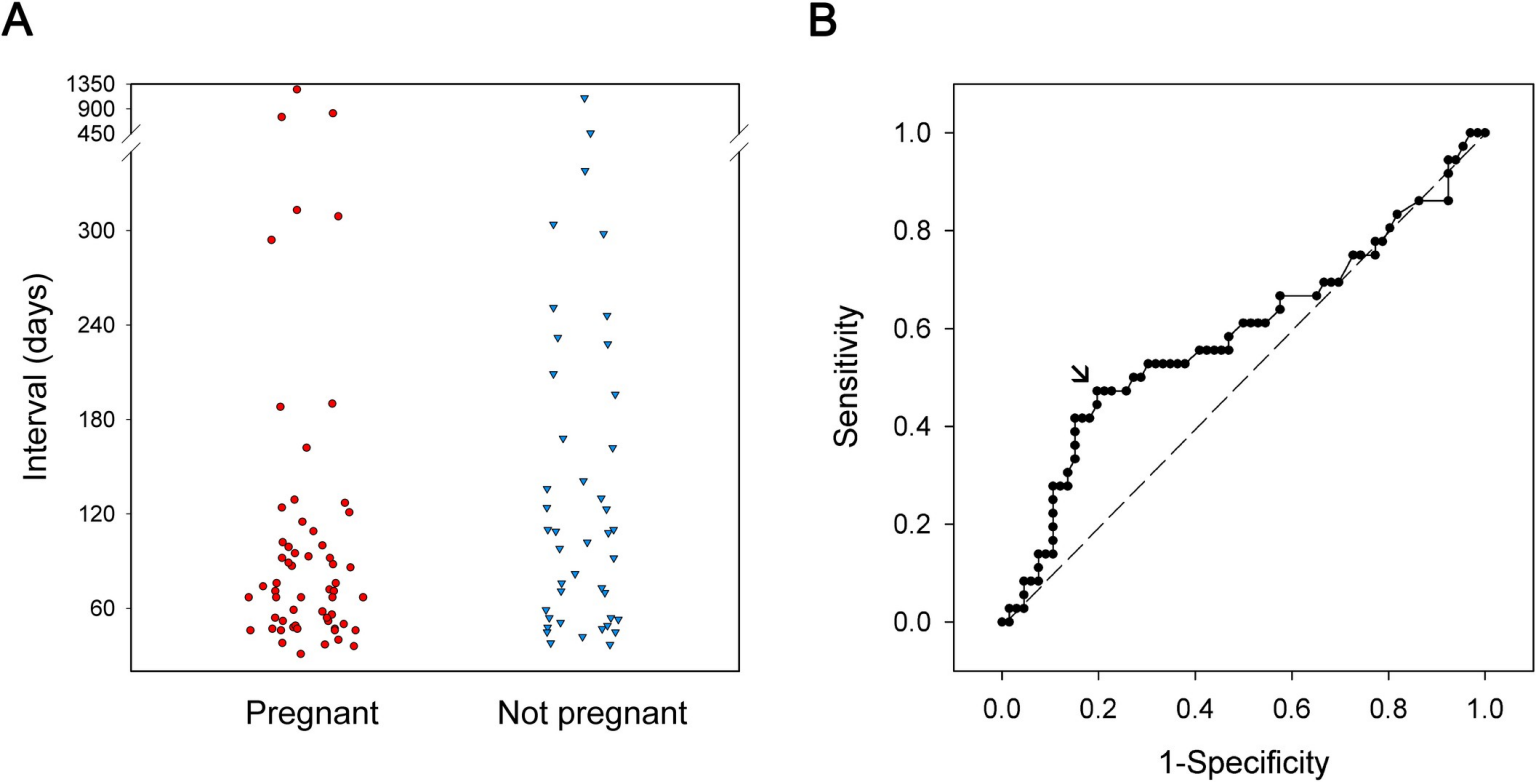
Interval of HSC-P to FET and biochemical pregnancy. Scatter plot of the intervals between HSC-P and FET, stratified by biochemical pregnancy outcome (A). The ROC curve for the probability of achieving chemical pregnancy at each different interval between HSC-P and FET (B). The point with the greatest trade-off between sensitivity and specificity, as determined by the Youden index, is at an interval of 120 days (AUC = 0.585, SE = 0.063, 95% CI from 0.462 to 0.708).

A scatter plot of the intervals between HSC-P and FBT, stratified by biochemical pregnancy, is shown in Fig 1A. There is a trend toward increased aggregation of positive biochemical pregnancies within 120 days. From the ROC curve (Fig 1B), it was determined that the best cut-off interval was 120 days, based on the Youden index, which maximizes the sum of sensitivity and specificity for pregnancy after HSC-P and FBT. At this cutoff, the sensitivity is 0.472 and specificity is 0.803. However, HSC-P by itself is not a good indicator of biochemical pregnancy, as judged by the AUC of just 0.585.

**Fig 2:**
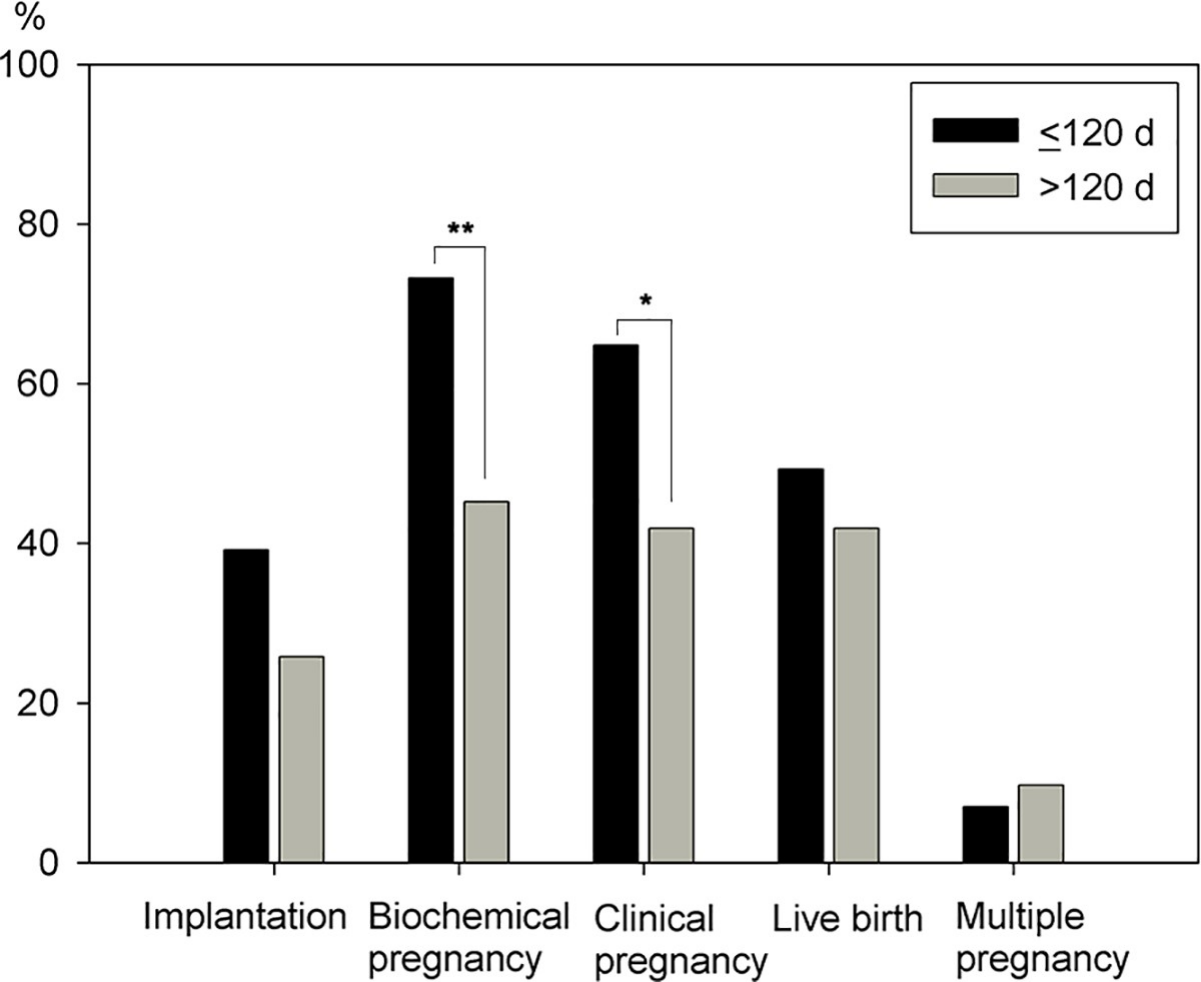
Pregnancy outcomes of frozen-thawed blastocyst transfer grouped by a cutoff of 120 days between HSC-P and FET. There is a significant increase in biochemical pregnancy rate and clinical pregnancy rate in those who received FBT within 120 days. ** *P*< .01; * *P*< .05.

A subgroup analysis of group 1 (Table 2) compared patients who received FBT within 120 days to those who received FBT later than 120 days. Both groups had similar baseline parameters, with the exception of a higher peak E2 and a larger number of retrieved oocytes during OPU in patients with intervals greater than 120 days. The numbers of endometrial polyps were not significantly different between the groups. The pregnancy outcomes between these groups are illustrated in Fig 2, where a significant higher biochemical pregnancy rate (73.2%, 95%CI = 62.9%-83.5%, 45.2%; 95%CI = 27.7%-62.7%, OR 3.3; *P* = .007), and clinical pregnancy rate (64.8%, 95%CI = 53.7%-75.9%, 41.9%; 95%CI = 24.5%-59.3%, OR 2.54; *P* = .032) are seen in those who received FBT within 120 days. There are no differences in implantation rate (39.2%, 95%CI = 31.5%-46.9%, 25.8%, 95%CI = 15.2%-36.4%; *P* = .056), live birth rate (49.3%, 95%CI = 37.7%-60.9%, 41.9%; 95%CI = 24.5%-59.3%, OR 1.34; *P* = .495) and multiple pregnancy rate (7.0%, 95%CI = -1.24%-2.64%, 9.7%; 95%CI = -0.718%-20.1%, OR 0.71; *P* = .650).

**Table 2:** Subgroup analysis of group 1 (polypectomy group).

	≤120 days	>120 days	^a^*P* value
Case no.	71	31	
Age (years)	36 (34, 39)	36 (33, 39)	.501
Gravidity	0 (0, 1)	0 (0, 1)	.363
Parity	0 (0, 0)	0 (0, 0)	.979
BMI (kg/m^2^)	22.2 (20.6, 23.8)	21.9 (20.9, 23.7)	.655
Polyp no.	2 (1, 4)	2 (1, 4)	.995
**Endometrial preparation**			.073
Natural cycle	21 (29.6%)	10 (32.3%)	
Artificial cycle	50 (70.4%)	21 (67.7%)	
**Endometrial thickness** (mm)	10.5 (9.5, 12)	11 (8.8, 12.2)	.990
**ET no.**	2 (2, 2)	2 (2, 2)	.759
**Slow-freezing/vitrification**	18 (25.4%) /53 (74.6%)	9 (29.0%) /22 (71.0%)	.698
**Good embryo no.**	1 (0, 2)	1 (0, 2)	.574
**OPU cycle**			
Peak E2 (pg/mL)	2630 (1624, 4358)	3670 (2513, 5515)	.009
Oocyte no. retrieved	14 (12, 19)	19 (12, 28)	.026
**Indication of IVF/ICSI**			.669
Male	29 (40.8%)	14 (45.2%)	
Ovarian	17 (23.9%)	4 (12.9%)	
Tubal	7 (9.9%)	4 (12.9%)	
Endometriosis	5 (7.0%)	1 (3.2%)	
PCOS	3 (4.2%)	3 (9.7%)	
Unexplained	10 (14.1%)	5 (16.1%)	

Data are presented as median (Q1, Q3) and n (%). BMI: body mass index. E2: estradiol. ET: embryo transfer. No.: number. OPU: ovum pick-up. IVF: in vitro fertilization. ICSI: intracytoplasmic sperm injection. PCOS: polycystic ovary syndrome

^a^*P* values were calculated using the Mann-Whitney U test for continuous variables, and Fisher’s exact test for categorical variables.

## Discussion

In the field of assisted reproduction, all possible efforts are made to optimize the conditions of the oocyte, sperm and endometrium. Endometrial thickness, duration of estrogen and progesterone priming, and endometrial receptivity arrays are just some of the parameters clinicians try to control in order to maximize endometrial receptivity [22–25]. Asymptomatic endometrial polyp is a commonly encounter problem, which may afflict 25% of women with unexplained infertility, and may only be detectable on hysteroscopy [26]. If these endometrial polyps are not diagnosed and adequately treated, fertility rates may be adversely impacted [27]. Expression of *HOXA10* and *HOXA11* are shown to be decreased in endometrial polyps, which may provide a molecular basis for the decrease in pregnancy rates [28]. Altered expression of the HOXA genes, which regulate endometrial development, has been suspected to be the culpable mechanism for the decreased implantation rates associated with endometriosis, polycystic ovarian syndrome, leiomyoma, adenomyosis, and hydrosalpinx [29–31]. Some researchers have even suggested routine screen of sub-clinical endometrial pathologies using office hysteroscopy in women with sub-fertile and women planning to receive assisted reproduction [32].

Previously, we found comparable reproductive outcome between patients who received HSC-P followed by FET and patients without endometrial polyp who underwent fresh ET [4]. Recently, with increased calls for the routine use of FET in order to obtain higher live birth rates [33], a re-examination of the effect of HSC-P with regards to the best interval between HSC-P and FET becomes necessary. FET does have the advantage of addressing some of the defects associated with fresh cycles, namely the risk of ovarian hyperstimulation syndrome, embryo-endometrium asynchrony, negative effects of premature progesterone elevation, and/or supraphysiologic estrogen [13, 34–36]. FET also allows time for preimplantation genetic testing and facilitates fertility preservation [37, 38]. Although FET does have its disadvantages, such as higher rates of hypertensive disorder during pregnancy, and large for gestational age [39, 40], more and more fertility centers worldwide are adopting the FET, which often accounts for more than half of the embryo transfer cycle performed.

In this retrospective study, we built on the results of our previous study [4] by enrolling an age-matched control group, and including patients who may have had a long interval between HSC-P and FBT. In this study, we found similar pregnancy outcomes between patients without endometrial polyp who received FBT, and patients who received HSC-P followed by FBT. In addition, there were significantly higher biochemical and clinical pregnancy rates when FBT was performed within 120 days of HSC-P. This could be due to a combination of the corrected uterine pathology, and a potentially beneficial effect induced by the endometrial trauma of HSC-P, similar to those of endometrial scratching [41–44]. However, the extent of this beneficial effect is uncertain, as some researchers have suggested that the benefits of endometrial scratching are lost after one menstrual cycle [45, 46]. In addition, a prospective controlled trial demonstrated that when endometrial scratching was performed at the time of OPU or concurrent to the embryo transfer cycle, decreased pregnancy rates have resulted [47, 48]. Based on these results, we think the effects of endometrial scratching may display a bimodal pattern. It may initially disturb the endometrium and causes harm to embryo implantation, but the secondary inflammation, which induces angiogenesis, may be beneficial to implantation. The effects of this enhanced implantation may persist for months, and have been attributed to monocyte recruitment to the injured sites. These monocytes are relative long-lived and can differentiate into resident macrophages/dendritic cells in response to cytokines expressed during implantation [49]. This has been seen in a murine model of epithelial injury where VEGF expression gradual increases 1 day after injury, significantly elevates from 3 to 5 days, and then plateaus between 7 and 14 days [50]. Whether these factors are the main contributing factors of endometrial scratching remain to be seen.

The fact that a better pregnancy outcome was achieved in cycles where FBT was performed within 120 days of polypectomy should not be of surprise. According to our previous work, 86% of wounds associated with HSC-P healed within 1 month, as seen by office hysteroscopy [5]. Follow-up durations of more than 1 year was associated with increased risks of polyp recurrences. The odds ratios of polyp recurrence between 1 and 2 years, between 2 and 3 years, and ≥ 3 years were 1.27, 2.33, and 3.92, respectively, when compared to < 1 year [7]. However, this outcome contrasts with other studies [8, 9], where no change in pregnancy outcome was seen in intervals below and above 6 months, or intervals of 1, 2 to 3, and greater than 3 menstrual cycle. There were limitations to those studies, however, which may impact its applicability. In the cohort study reported by Eryilmaz et al., they excluded analysis of patients with multiple endometrial polyps [8]. In addition, although the reported average age of 31 years was younger than normally encountered, the pregnancy outcome were subpar by current standards (fertilization rate 41 to 43%, clinical pregnancy rate 20 to 21%, and no reported live birth rate) [8]. In the cohort study by Pereira et al., the evaluated intervals were small, with the maximum interval extending no longer than 5 menstrual cycles after HSC-P [9]. In addition, there were no report of embryo grades, which preclude direct comparison of embryo factors [9]. Lastly, marked heterogeneity existed in both studies as they used a combination of cleavage and blastocyst stage embryos in the fresh transfers.

In this study, we only studied FET cycles and transferred embryos exclusively at the blastocyst stage, which reduced the heterogeneity of the embryo factors. All reported cases are from the patients’ first FBT, which should control biased due to selection of patients with recurrent implantation failure and being their best shots. Therefore, the results should adequately reflect the relationship between HSC-P and FET, and gives a valid estimate of the appropriate interval between HSC-P and FET. Our study results agree with another retrospective cohort study, where a trend toward higher pregnancy rate in fresh and vitrified-warmed blastocyst transfers was seen when FET was performed closer to the hysteroscopic procedures [51].

In our studied cases, 19 patients underwent embryo transfer more than 6 months after HSC-P, and 5 patients waited for more than a year. We investigated the reasons behind this prolonged embryo transfer deferral, and we discovered that most were trying to conceived naturally or via other forms of assisted reproductive techniques, such as ovulation induction with oral and parenteral medications, timed intercourse, and intrauterine insemination. Others reported being concerned about the potential adverse effects of ovarian stimulation or HSC-P on fetal development, and wished to postpone FET to minimize any conceivable effect on uterine environment [52]. In summary, all cases were due to patient preference rather than medical advice.

As with all clinical studies, this study has limitations which deserve mention. A major limitation is the retrospective nature of the study. Even though we matched the age factor and the groups were generally the same with similar gravidity, parity, BMI, number of endometrial polyps and methods of endometrial preparation, the possibility of an unknown confounding cannot be completely excluded. For example, it is unknown if the higher estradiol, and the larger total number of retrieved oocytes in those with interval >120 days significantly affected the subsequent embryo quality in the frozen-thaw cycles. However, we do not think so as the current literature does not suggest a lasting effect from differences in estrogen production on embryo quality [53, 54]. In addition, the AUC of the constructed ROC was low, which could reflect the fact that endometrial polyp is only one of many determining factors of endometrial receptivity. Before being validated by future studies, we should also adopt this 120-day cutoff with caution. Lastly, this study may be underpowered to examine the secondary outcomes, such as live birth rates and multiple pregnancy rates. The study may also not be applicable to cleavage-stage embryos and PGT-tested embryos, as it was exclusively performed on FBT.

From this study, we suggest patients not waiting longer than 4 months after hysteroscopic polypectomy to undergo frozen-thaw embryo transfer. Extending the interval between HSC-P and FBT beyond 120 days may be associated with decreased pregnancy rates, which could be due to polyp recurrence or diminishing benefits from traumas induced by HSC-P, similar to endometrial scratching. Further studies are needed to validate the pathophysiological aspects of these effects.

## Supporting information

S1 File Data of all patients. The excel file is our original data of all 204 patients, including their basic characteristics, endometrial, oocyte, and embryo conditions, and pregnancy outcomes.
(XLS)
